# Striatal Hypodensities, Not White Matter Hypodensities on CT, Are Associated with Late-Onset Depression in Alzheimer's Disease

**DOI:** 10.4061/2011/187219

**Published:** 2011-09-21

**Authors:** Jessica A. Brommelhoff, Bryan M. Spann, John L. Go, Wendy J. Mack, Margaret Gatz

**Affiliations:** ^1^Department of Psychology, University of Southern California, Los Angeles, CA 90089, USA; ^2^Department of Neurology, University of Southern California, Los Angeles, CA 90033, USA; ^3^Department of Radiology, University of Southern California, Los Angeles, CA 90033, USA; ^4^Department of Preventive Medicine, University of Southern California, Los Angeles, CA 90089, USA; ^5^Department of Medical Epidemiology and Biostatistics, Karolinska Institutet, 17177 Stockholm, Sweden

## Abstract

This study examined whether there were neuroanatomical differences evident on CT scans of individuals with dementia who differed on depression history. Neuroanatomical variables consisted of visual ratings of frontal lobe deep white matter, subcortical white matter, and subcortical gray matter hypodensities in the CT scans of 182 individuals from the Study of Dementia in Swedish Twins who were diagnosed with dementia and had information on depression history. Compared to individuals with Alzheimer's disease and no depression, individuals with Alzheimer's disease and late-onset depression (first depressive episode at age 60 or over) had a greater number of striatal hypodensities (gray matter hypodensities in the caudate nucleus and lentiform nucleus). There were no significant differences in frontal lobe deep white matter or subcortical white matter. These findings suggest that late-onset depression may be a process that is distinct from the neurodegenerative changes caused by Alzheimer's disease.

## 1. Introduction

While some studies have suggested that a history of depression is a risk factor for dementia later in life (e.g., [1, 2]), a number of authors have concluded that depression is an early symptom of dementia [3–6]. Boland [7] proposed that prodromal depression arises from dementia-related neuropathology. Several studies of nondemented older adults have suggested an association between white matter lesions and depression, specifically late-onset depression [8–14]. In a longitudinal study, white matter changes pre-dated and independently predicted the onset of depressive symptoms in older adult participants [15], providing some evidence that white matter changes are an antecedent to depression. Furthermore, in their review, Schweitzer et al. [16] concluded that the white matter changes that are common in individuals with late-onset depression were associated with cognitive impairment, and thus, were indicative of a prodrome to dementia. 

A greater amount of total brain white matter lesions has been associated with more severe depression or a greater number of depressive symptoms [9, 17, 18]. Other studies have suggested that white matter lesions in the frontal lobe are specifically associated with a higher rate of depressive symptoms among persons without dementia [8, 19]. A review of white matter lesions and clinical manifestations concluded that while periventricular white matter lesions are often associated with Alzheimer's disease (AD), lesions in the subcortical white matter are more often associated with late-onset depression [16]. Among 39 hospital inpatients over age 60 with severe depression, later age of first depressive episode was associated with a greater severity of subcortical deep white matter hyperintensities [20]. After a mean followup of 14 months, 27% of the original 39 inpatients had developed a probable dementia syndrome, which was predicted by a later age of depression onset and subcortical white matter hypodensities (WMH) [21]. 

In their review of structural brain abnormalities in affective disorders, Taylor and Krishnan [22] posit that for late-onset depression “evidence is strongest for a contributory effect of [subcortical hyperintensities], particularly as to hyperintensities of the basal ganglia” (page 61). In fact some of the earliest studies in this area dating back nearly 20 years noted the greater prevalence and severity of subcortical hyperintensities among depressed older adults. For example, Coffey et al. [23] found lesions in the subcortical gray matter nuclei (basal ganglia and thalamus) to be significantly more common in depressed than in nondepressed older adults (60 years of age and older). Greenwald et al. [24] sought to further specify the location of the subcortical lesions associated with late-life depression, comparing the subcortical gray matter hyperintensities in a group of older adults receiving treatment for depression to a group of older adult community controls. In this sample, left-hemisphere hyperintensities in the putamen, a part of the lenticular nucleus, which is a component of the striatum, were significantly more common among depressed individuals. 

These findings of an association between frontal lobe, subcortical deep WMH, and striatal hypodensities and depression suggest that damage to frontal-subcortical circutry may be responsible for late-onset depression. Such damage would be consistent with the frontostriatal hypothesis of depression [25, 26], which suggests that damage to the frontal lobe and striatum (input nuclei for the basal ganglia comprised of the caudate nucleus and the putamen) are associated with depression, though the exact nature of the association remains unclear. In her review focusing on subcortical ischemic vascular dementia, Chui [27] posits that deep white matter lesions can disrupt frontal-subcortical loops and the white matter tracts therein, which are important for cognition and emotion. 

Increasing evidence suggests that the pathenogenesis of white matter lesions may be largely due to cerebrovascular disease (CVD). One study [28] indicated that cardiac insufficiency, one of the sequelae of cardiovascular disease, results in hypoperfusion (decreased blood flow) to subcortical regions of the brain. Frontosubcortical loops and the associated white matter tracts seem to be especially vulnerable to hypoperfusion and ischemia [27]. Thus, even in the absence of a stroke, white matter changes could occur secondary to CVD-related systemic hypoperfusion and ischemia. In conjunction with the vascular depression hypothesis [29], CVD may be considered as a potentially important antecedent in the relationship between late-life depression and white matter pathology.

The aim of the present study was to evaluate neuroanatomical alterations on the CT scans of individuals with dementia based upon a history of late-onset depression (LOD, first episode of depression at age 60 or older) or late-life depression (LLD, any episode of depression at age 60 or older). We hypothesized that individuals with dementia and LOD and/or LLD would be more likely than non-depressed individuals with dementia to exhibit hypodensities in the frontal lobe deep white matter and subcortical white matter (subinsular region and internal capsule), as well as the subcortical striatal gray matter (caudate nucleus and lentiform nucleus, which includes the putamen and globus pallidus) on CT scans. We further hypothesized that these patterns would be evident among all demented individuals and in Alzheimer's disease alone. 

## 2. Methods

### 2.1. Participants

 Study participants were part of the Study of Dementia in Swedish Twins (HARMONY) [30]. The HARMONY study population included all twins in the Swedish Twin Registry [31] who were aged 65 and older and alive during the telephone-screening phase. Although this study uses data from Swedish twins, it is not a twin study per se.

Dementia was ascertained through a two-step procedure that entailed an initial cognitive screening with a subsequent diagnostic workup of each suspected case. In brief, the TELE [32, 33] and the Blessed Dementia Rating Scale (BDRS) [34] were used to screen for cognitive dysfunction. Twins who screened positive for suspicion of dementia (and their twin partners) were evaluated in person by a physician and nurse. Final diagnoses of dementia were determined by a multidisciplinary consensus board. Dementia was diagnosed according to the criteria in the Diagnostic and Statistical Manual of Mental Disorders IV [35] and differentially diagnosed for AD versus vascular dementia using NINCDS/ADRDA [36] and NINDS-AIREN criteria [37]. The HARMONY study used two sources of information to estimate the age of dementia onset: informant reporting during an in-depth semistructured interview and medical records [38].

**Figure 1:**
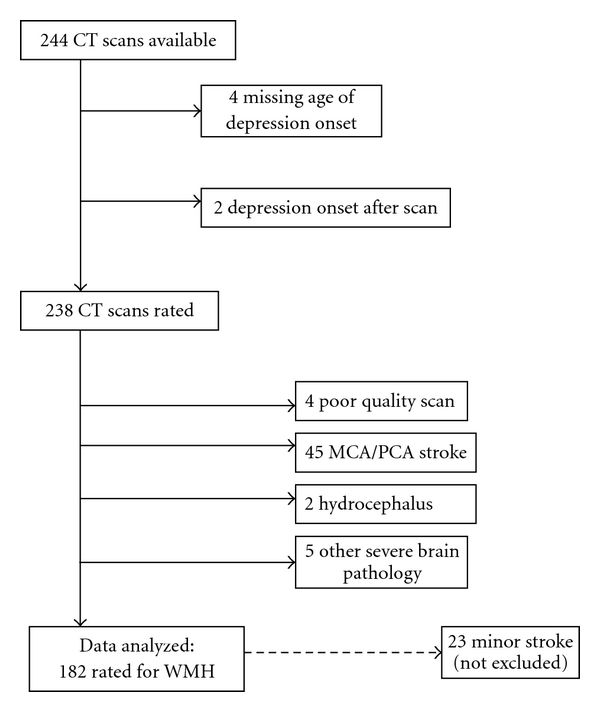
Study sample.

The sample for the present study (*N* = 238) consisted of all HARMONY twins who (1) were diagnosed as having dementia, (2) had a hard copy of their CT scan (performed as part of the clinical phase of dementia assessment) that could be assessed by the CT raters, and (3) did not meet any of the exclusion criteria (see Figure 1). Exclusion criteria included missing information on timing of depressive episodes (*N* = 4) or first depression onset more than six months after the scan date (*N* = 2). 

Of the 238 CT scans scored by the raters, 56 were excluded during the rating process. Four scans were excluded due to the poor quality of the scan, 45 because there was evidence of major stroke (i.e., middle cerebral artery stroke, posterior cerebral artery stroke), 2 because of hydrocephalus, and 5 because of other major brain problems (e.g., evidence of major brain surgery or traumatic brain injury). Those with evidence of a minor stroke (i.e., small lacunar infarct) were not excluded. The final sample included white matter hypodensity and striatal hypodensity ratings for 182 individuals.

### 2.2. Neuroimaging

Diagnostic neuroimaging utilized CT scans because at the time of the clinical diagnostic assessment insufficient numbers of participants lived close to MRI centers. Twins completed CT scans at their most convenient participating CT center. All CT technicians were given standardized instructions to perform a CT of the brain with standard slices and noncontrast enhancement. The slices were to be four to five millimeters from the base of the skull and eight to ten millimeters from the pars petrosa ossis temporalis. Contrast enhancement was performed if needed for clinical reasons. The diagnostic protocol allowed individuals who had a CT within six months prior to the clinical workup to provide a copy of that scan instead. After obtaining a clinical read, CT scans were deidentified and made available for research purposes.

### 2.3. Visual Rating of CT Scans

 B.M.S. and J.L.G. served as the CT raters. Raters were blind to clinical diagnosis and any demographic information, including which scans were from twin pairs, as well as the age, gender, and zygosity of the scanned individual. If either rater determined that an image quality was unacceptable, it was excluded from the analyses. If there was evidence of a major stroke, such as middle cerebral artery or posterior cerebral artery stroke, the CT was not included in the analyses. Individuals with small lacunar infarcts, however, were not excluded. CT scans that indicated hydrocephalus or other severe neuropathology, such as evidence of a brain tumor, major brain surgery, or traumatic brain injury, were also excluded from the analyses. 

#### 2.3.1. Frontal Deep White Matter Hypodensities

 Deep white matter hypodensities in the frontal lobe were rated on a modified version of the Age-Related White Matter Changes (ARWMC) Scale [39], where 0 = an absence of hypodensities, 1 = one focal hypodensity (≥5 mm), 2 = more than one focal hypodensity, 3 = confluent hypodensities, and 4 = confluent hypodensities with additional discrete focal hypodensities. Focal hypodensities were defined as discrete hypodensities greater than five millimeters in size. Confluent hypodensities were present when discrete hypodensities could not be separately defined. Deep white matter hypodensities (DWMH) were defined as WMH that were located medially from the sulci. The ratings of frontal DWMH in the right and left hemisphere were highly correlated (Spearman's rho = 0.92, *P* < 0.0001). Therefore, summary scores for frontal DWMH used the maximum score for the right and left hemispheres.

#### 2.3.2. Subcortical White Matter Hypodensities

 The density of the white matter in the internal capsule and subinsular region (comprised of the external capsule, claustrum, and extreme capsule) was compared to the density of homogenous areas of white matter in the frontal lobe to determine if there were relative hypodensities evident in either of these components. The location, side (left versus right hemisphere), and rating of the hypodensities within these regions were indicated, with hypodensities rated on the modified version of the ARWMC Scale. The ratings of subcortical WMH in the right and left hemisphere were significantly correlated (Spearman's rho = 0.79, *P* < 0.0001); a summary subcortical WMH score was created using the maximum score for the right and left hemispheres.

#### 2.3.3. Basal Ganglia-Thalamic Hypodensities

 The raters also examined each scan for gray matter hypodensities in the basal ganglia and thalamic regions. The homogeny of the gray matter in the striatum (caudate nucleus and lentiform nucleus), substantia nigra, and thalamus were compared to one another to identify hypodensities. The total number of hypodensities in the striatum on both right and left hemispheres was summed.

#### 2.3.4. Interrater Reliability

 Each CT scan was scored separately by both raters. Interrater reliability for bilateral WMH was substantial (weighted kappa, right WMH = 0.92; weighted kappa, left WMH = 0.89) and slightly lower though adequate for bilateral basal ganglia-thalamic hypodensity ratings (weighted kappa, right = 0.73; weighted kappa, left = 0.62). In cases of disagreement, the scans were rerated conjointly, and the consensus rating was used for analysis.

### 2.4. Depression

History of depression, comorbid depression, and estimated age of depressive episodes were determined using information from four sources: (1) the national computerized Inpatient Discharge Registry (IDR), (2) the national registry of inpatient psychiatric hospital services, (3) medical history provided by an informant, and (4) medical records.

The Swedish Twin Registry is linked to the national computerized Inpatient Discharge Registry that records all inpatient hospital discharges in Sweden. Discharge diagnoses use International Classification of Disease (ICD) codes. If the discharge date was prior to 1969, the ICD-7 coded depression diagnoses as 302 (involutional melancholia), 314 (depressive neurosis), and 790.2 (other recurrent depressive disorder). If the discharge date was 1969–1986, the ICD-8 coded depression diagnoses as 296.0 (involutional melancholia), 298 (reactive depressive psychosis), 300.4 (depressive neurosis), and 790.2 (other recurrent depressive disorder). If the date of discharge was 1987–1996, the ICD-9 coded depression diagnoses similar to the ICD-8, with 296.2 (depressive psychosis), 296.3 (recurrent depressive psychosis), 296.82 (atypical depression), 300.4 (dysthymia), and 311 (depression, NOS). From 1997, the ICD-10 depression diagnoses included F32 (depressive episode), F33 (recurrent depressive disorder), and F34.1 (dysthymia). There were 11 participants with discharge diagnoses of depression in the Inpatient Discharge Registry. 

The Swedish Twin Registry has also been linked to a national registry of inpatient psychiatric hospital services that was maintained between 1967 and 1983. For each person entered in this registry, there is a record of the discharge diagnosis and the date of hospitalization. All diagnoses are given in terms of an ICD-8 diagnosis (see above). There were two participants with diagnoses of depression in the inpatient psychiatric hospital services registry, both of whom also had a diagnosis of depression in the Inpatient Discharge Registry.

Thus, a total of 11 participants in this study had at least one depression-related discharge diagnosis in the IDR between the years of 1964 and 2004, a span of 40 years, or between 1967 and 1984 in the inpatient psychiatric hospital services registry. The most common depression-related discharge diagnosis was depression not otherwise specified, followed by dysthymia, recurrent depressive disorders, and depressive episodes of mild, severe, or other characteristics. Discharge diagnoses in both the IDR and the psychiatric hospital discharge registry that were regarded as not depression-related included bipolar affective disorder and manic-depressive reaction, manic or unspecified type, schizoaffective disorder, and unspecified mood disorders. 

Medical history reported by an informant was collected during the clinical evaluation. The history included whether the individual had any history of “major depressive disorder” or “reactive depression,” and if so, the date or dates of onset. The first depressive episode according to the medical history used the earliest date recorded for the onset of a depressive disorder and the most recent episode of depression used the latest date recorded for a depressive episode. 

Medical records, ordered during the clinical evaluation phase, were coded by the assessment team to reflect whether the twin had been diagnosed with depression. Records typically go back approximately ten years before the clinical evaluation for dementia. Thus, these records were most helpful in determining whether late-life depression was present. Data included the onset and dates of depression. 

Use of antidepressant medications was also coded from the medical records and medical history. A total of 31 individuals were prescribed antidepressant medication, but did not have any other information indicating that they had ever received a diagnosis of depression. These individuals were coded as not having a history of depression, as it is possible that these medications were prescribed for reasons other than depression.

The age of the first episode of depression was determined using the age at the earliest reported occurrence of depression across all sources. Individuals with a first episode of depression at age 60 or older were considered to have late-onset depression (LOD). Individuals who had prior episodes of depression including at least one episode of depression at age 60 or over were considered to have a history of late-life depression (LLD). In sum, a total of 132 (72.5%) individuals did not have a history of a depression diagnosis and 50 (28.5%) individuals had a history of a depression diagnosis. Of these individuals, 36 had LOD, having their first depressive episode after age 60, with a mean (SD) age of first depressive episode of 74.2 (7.0) years. Of the 14 individuals who had their first depressive episode before age 60, nine individuals had an episode of depression occurring after age 60. Thus, 45 of the 182 participants were considered to have LLD. Those with an early episode of depression that did not recur in later life (*N* = 5) were not included in further analyses.

### 2.5. Cerebrovascular Disease

 Cerebrovascular disease risk factors, as indicators of CVD risk, were examined as potentially important covariate. Data on hypertension, diabetes, atrial fibrillation, peripheral artery disease, transient ischemic attack, and coronary artery disease (CAD) indicators were extracted from the participant's medical records and coded by the assessment team to reflect whether the individual had a history of any of these risk factors. Coronary artery disease was considered present if the individual had a history of myocardial infarction, angina, or heart failure.

### 2.6. Other Covariates

 Additional covariates included age at the time of the CT scan, dementia duration (i.e., age at the time of the scan compared with age of dementia onset), gender, zygosity, and number of years of education completed. Because of the cross-sectional study design, particular attention was paid to the duration of dementia at the time of the CT scan (calculated by subtracting age of dementia onset from the age at the CT scan).

### 2.7. Analyses

 Associations between history of LOD (no depression versus LOD) and all potential demographic and medical confounders were initially examined to determine factors to include as covariates in multivariate models. Chi-square tests were used to examine whether gender, zygosity, or risk factors for CVD (hypertension, diabetes, atrial fibrillation, peripheral artery disease, transient ischemic attack, and coronary artery disease indicators) differed by history of LOD. One-way ANOVA was used to determine whether age at CT scan, age of dementia onset, duration of dementia at the time of the CT scan, or level of education differed by presence versus absence of LOD. 

Kruskal-Wallis chi-square was used to examine whether frontal deep white matter or subcortical white matter score varied by age at CT scan or dementia duration. Fisher's Exact Test was used to determine whether frontal deep white matter or subcortical white matter score differed by gender. Simple linear regression was used to test whether number of striatal hypodensities varied by age, gender, or dementia duration. We took a conservative approach to determining which potential confounders should be included in the final model, including covariates with an association of *P* < 0.15.

Logistic regression for dichotomous outcomes controlling for gender and history of TIA was used to examine the association between LOD and the presence of any frontal lobe deep WMH (focal and confluent WMH versus no WMH), as well as between LOD and the presence of confluent frontal lobe deep WHM (confluent WMH versus no WMH and focal WMH only). To analyze the relationship between LOD and level of subcortical WMH (0 = no WMH, 1 = one WMH, 2 = more than one WMH, 3 = confluent WMH), ordinal logistic regression was used, controlling for age at CT scan and zygosity. The association between numbers of striatal hypodensities and LOD was examined using one-way ANOVA; there were no demographic or medical covariates associated with striatal hypodensities. All of the analyses testing white matter associations with LOD were reanalyzed using LLD. Finally two sets of analyses were performed, the first using all participants with dementia and the second using only individuals with Alzheimer's disease.

Seven complete twin pairs were included in these analyses. To account for the possibility that these seven pairs had correlated data, we also used generalized estimating equations (GEE) to examine the association between the presence of frontal deep WMH and LOD, the amount of subcortical WMH and LOD, and the number of striatal hypodensities and LOD for both the total sample and the Alzheimer's disease only sample. GEE accounts for the lack of independence between twin observations.

## 3. Results

The most frequent type of dementia among these 182 individuals was Alzheimer's disease (*N* = 127, 69.8%), followed by vascular dementia (*N* = 29, 15.9%) and dementia not otherwise specified (*N* = 16, 8.8%). An additional five participants (2.7%) were diagnosed with dementia of a mixed type (both Alzheimer's disease and vascular dementia pathology) and another five (2.7%) individuals had other forms of dementia (e.g., frontotemporal dementia). Average duration of dementia (from dementia onset to date of CT scan) was 5.2 years (SD = 4.25, range 0–23 years). The majority of participants were female (*n* = 116, 63.7%) and the average age at CT scan was 80.6 years (SD = 6.7 years, range 56–96 years). Participants had 7.4 years (SD = 2.4) of education on average, and 51 participants (28.0%) were from a monozygotic twin pair. Demographic characteristics are presented in Table 1 for all dementias and for Alzheimer's disease alone. 

### 3.1. Associations between Imaging Data and Sample Demographic Characteristics

 Across all study participants, 39.6% (*N* = 72) had at least one hypodensity in the frontal lobe deep white matter and 51.6% (*N* = 94) had at least one subcortical white matter hypodensity. Among the 94 participants with subcortical white matter hypodensities, the majority (*N* = 88, 93.6%) had at least one hypodensity in the subinsular region, only six individuals had hypodensities solely within the internal capsule, and 30.8% (*N* = 29) had hypodensities both in the subinsular region and the internal capsule. 

Age at the time of the scan, duration of dementia, and education were not associated with frontal deep white matter hypodensities. Male gender was associated with the presence of frontal deep white matter hypodensities (53.0% of males versus 34.5% females; *P* = 0.01); gender was therefore included as a covariate in the multivariate logistic regression analysis. Zygosity was not associated with presence of frontal lobe deep WMH. 

Age at the time of the CT scan was significantly associated with subcortical white matter hypodensities, such that older individuals had more subcortical WMH (*χ* ^2^ = 1.59, df = 3, *P* = 0.03). Individuals who were monozygotic twins were less likely than nonmonozygotic twins to have subcortical WMH (37.2% of monozygotic twins; 57.2% of dizygotic twins; *χ* ^2^ = 10.43, df = 3, *P* = 0.02). Gender, education, and duration of dementia were not associated with subcortical white matter hypodensities. Thus, age at the time of the CT scan and zygosity were included as covariates in the multivariate ordinal logistic regression models. 

Across all study participants, 30.8% (*N* = 56) had at least one hypodensity in the striatum. A simple linear regression analysis indicated that age at the time of the scan, education, and the duration of dementia were not associated with the number of striatal hypodensities. Gender and zygosity were also not associated with striatial hypodensities.

### 3.2. Associations between Imaging Data and Vascular Risk Factors

 Presence of frontal deep WMH, level of subcortical WMH severity, and total number of striatal hypodensities did not significantly vary by history of CAD. History of a TIA was present for 14 individuals in the nondepressed group and no individuals with a history of depression. Controlling for gender, individuals with a history of TIA were 3.92 (95% CI = 1.16, 13.24, *P* = 0.03) times more likely than individuals without a history of TIA to have hypodensities in the frontal lobe deep white matter. Amount of subcortical WMH and striatal hypodensities did not significantly vary by history of TIA.

### 3.3. Associations between Imaging Data and Dementia Type

 A logistic regression model controlling for gender indicated that individuals with vascular dementia were 2.67 times more likely to have frontal lobe deep WMH than individuals with other types of dementia (95% CI = 1.24, 5.76, *P* = 0.012). Gender also remained significant, and men were 1.85 times more likely to have frontal lobe deep WMH than women (95% CI = 1.08, 3.61, *P* = 0.025). Although individuals with vascular dementia were more likely to have a higher ranked amount of subcortical WMH, this association was not statistically significant (*P* = 0.14). Presence of striatal hypodensities did not differ according to dementia type.

### 3.4. Depression Status

Individuals with no history of depression and individuals with LOD did not differ significantly on most demographic variables (Table 2). Similarly, no differences were observed between the non-depressed and the LLD groups. Participants in the LOD group were more likely to be a member of a monozygotic twin pair compared to non-depressed participants (*P* = 0.05). Gender and years of education did not significantly differ by either history of LOD or history of LLD. 

Among all study participants, the mean (SD) duration of dementia at the time of the CT scan was 5.2 (4.2) years. There was no significant difference between the no depression and the LOD group with respect to duration of dementia at the time of the CT scan. At the time of the CT scan, those with LOD had an average dementia duration of 4.2 years (SD = 3.5) and individuals with no history of depression had an average dementia duration of 5.2 years (SD = 4.2). 

The presence of CAD and TIA differed by depression group (Table 2). Individuals in the non-depressed group were more likely to have had a transient ischemic attack than individuals in the LOD group (*P* = 0.04). Individuals with LOD were more likely than non-depressed individuals (RR = 1.43, 95% CI = 0.92, 22.33, *P* = 0.13) to have a history of CAD. CAD was not significantly more prevalent, however, in the LLD group (40.0%) compared to the no depression group (31.1%; *P* value for difference = 0.27).

### 3.5. White Matter Hypodensities and LOD/LLD

 Among individuals with LOD, if there were hypodensities in the frontal lobe deep white matter, they were generally rated as confluent (92.9% of deep WMH were rated as confluent). Therefore, we examined whether LOD was associated with confluent hypodensities, testing frontal deep WMH (DWMH) as a dichotomous variable—confluent frontal lobe DWMH versus nonconfluent (no or only focal hypodensities) frontal lobe DWMH. Controlling for gender, LOD was not significantly associated with a greater risk of confluent frontal lobe DWMH for both the total dementia and Alzheimer's disease only analyses, although the pattern of the results was in the hypothesized direction (Table 3). A similar pattern was observed for LLD, where, after controlling for gender, the association between LLD and risk of confluent frontal lobe DWMH was nonsignificant for both the total dementia (OR = 1.38, 95% CI = 0.66, 2.88) and Alzheimer's disease only (OR = 2.12, 95% CI = 0.86, 5.28). There was no association between frontal lobe DWMH dichotomized as presence versus absence of hypodensities and dementia or AD (Table 4). 

After controlling for age at CT scan and zygosity, no association was observed between subcortical WMH and LOD. The distribution of subcortical WMH is shown in Table 5. Results were similar for LLD versus no depression comparison. A greater age at the time of the CT scan and a greater amount of subcortical WMH (OR = 1.08 per year of age, 95% CI = 1.01, 1.12, *P* = 0.006) were the only statistically significant association in the multivariate model.

### 3.6. Striatal Hypodensities and LOD/LLD

Number of striatial hypodensities is shown in Table 6. One-way ANOVA indicated significantly more striatal hypodensities for individuals with late-onset depression compared with non-depressed individuals when AD was considered alone. The patterns when comparing LOD to total dementia and when comparing LLD to non-depressed were similar (0.05 < *P* < 0.10). GEE was used to account for the possible correlation between twin pairs. The relationship between a greater number of striatal hypodensities and LOD remained significant (OR = 2.27, 95% CI = 1.02, 5.05).

### 3.7. Source of Depression Information

Despite the demonstrated concordance between medical records and informant-reported depression, we examined in a post hoc analysis whether the relationships between hypodensities and depression differed based upon the source of information about depression. There was no significant difference in the relationship between frontal DWMH, subcortical WMH, striatal hypodensities and depression when informant-reported medical history information was disregarded. There were also eight individuals missing either an informant report (*N* = 6) or coded medical records (*N* = 2). Dropping these individuals from our analyses did not affect the outcomes of our analyses.

## 4. Discussion

The aim of the present study was to investigate the underlying neural mechanisms linking depression and dementia, stemming from the idea that there could be a pathophysiological basis in the brain for the comorbidity of depression and dementia [1]. According to the fronto-striatal hypothesis of vascular depression, white and gray matter lesions in the fronto-striatal pathway resulting from cerebrovascular disease (as well as normal aging) may cause late-onset depression [25]. In line with the fronto-striatal hypothesis, we predicted that demented individuals with a LOD or a history of LLD would have a higher prevalence of WMH in the frontal lobe deep white matter and subcortical white matter, as well as a greater amount of gray matter hypodensities in the striatum, compared to individuals with dementia and no depression. While similar hypotheses have been tested among individuals with late-onset and/or late-life depression, they have rarely been considered among individuals with both dementia and late-onset and/or late life depression.

In support of one of the main hypotheses of the study, individuals specifically with Alzheimer's disease and late-onset depression had a significantly greater total amount of striatal hypodensities compared to individuals with Alzheimer's disease and no history of depression. Further analysis revealed that this association was not explained by any other medical or demographic covariate and remained significant even after accounting for the possibility that the twin pairs included in the study sample were not truly independent observations. According to the bivariate level analyses, there were no associations between striatal hypodensities and any of the demographic or medical history confounders. Only CAD differed between the LOD and non-depressed group in the individuals with AD (*χ* ^2^ = 3.89, df = 1, *P* = 0.048), with the LOD group more likely to have CAD than the non-depressed group (48.0% versus 27.4%, resp.). However, CAD was not associated with number of total striatal hypodensities. Thus, there were no other tested covariates that could explain the association we found between the LOD group and striatal hypodensities among the AD group. Although there was a consistent pattern of findings for striatal hypodensities, the finding was statistically significant only among those with Alzheimer's disease in particular, when comparing those with late-onset depression to those with no earlier history of depression. 

Although the direction of some of the associations was consistent with our hypotheses, the presence (versus absence) of frontal lobe deep WMH did not differ significantly between the non-depressed and LOD or LLD groups, nor did severity of subcortical white matter hypodensities differ between the nondepressed and LOD or LLD groups. Male gender and a history of TIA were the only two significant predictors of presence of frontal lobe deep WMH. While the prevalence of ischemic neuropathology on CT in cognitively intact patients with TIAs has been estimated at approximately 40%, most abnormalities are diffuse throughout the cerebral white matter or basal ganglia [40]. Therefore, our finding that hypodensities were specific to the frontal lobe deep white matter may be specific to individuals with dementia. Severity of subcortical WMH was only associated with age at CT scan. 

### 4.1. White Matter Hypodensities and LOD/LLD

 Our lack of statistically significant findings with respect to the white matter components of the fronto-striatal pathway was surprising in light of the solid base of literature supporting an association with late-onset and late-life depression. Several differences between the present study and past studies may explain this discrepancy. Most of the study populations that found the association between WMH and LOD and LLD were comprised of cognitively intact older adults or older adults with mild cognitive impairment. All of the individuals in the present study had dementia, with approximately 70% diagnosed with Alzheimer's disease. Therefore, this study at least partially “controls” for the neuropathology related to dementia. However, it could be that by the time cognitive and functional impairment has progressed to the point of dementia, the neurological damage caused by dementia is widespread enough to subsume the neuropathology of late-onset depression such that the two neuropathologies become indistinct. In fact, this may even happen in the early stages of dementia, as suggested by a study of mildly demented patients both with and without a subsyndromal level of depressive symptoms, which failed to find any significant relationship between white matter changes and depressive symptoms [41]. At variance with this, however, Lavretsky et al. [18] found that higher lacunar volume in the white matter was associated with neuropsychiatric symptoms (including depressed mood, anhedonia, anergia, and apathy) in a group of both cognitively intact and cognitively impaired individuals, even after controlling for cognitive status.

### 4.2. Subcortical Gray Matter Lesions and LOD in Alzheimer's Disease

 The finding that individuals with LOD and Alzheimer's disease had a greater number of striatal hypodensities compared to individuals without depression and Alzheimer's disease is consistent with several prior imaging studies of late-life depression in nondemented individuals demonstrating a greater prevalence of lesions in the putamen among individuals with geriatric depression [24] and reduced caudate nucleus volume among adults with depression [42]. The striatum represents the subcortical gray matter components of the fronto-striatal pathway. Thus, the finding that individuals with AD and LOD have a greater amount of striatal hypodensities compared to individuals with AD and no depression provides some support for the fronto-striatal hypothesis of depression among individuals with dementia. 

Although Alzheimer's disease is a “cortical” dementia, the individuals with LOD had a greater prevalence of hypodensities in the subcortical gray matter compared to the individuals with Alzheimer's disease and no depression. As subcortical changes are not typically associated with Alzheimer's disease, the greater amount of striatal hypodensities evident in the individuals with Alzheimer's disease and LOD may represent a neuropathological process associated with late-onset depression that is distinct from the neurodegenerative changes caused by Alzheimer's disease. In fact, dementia characterized by the neurodegeneration of subcortical structures, or subcortical dementia, is often associated with depression-like symptoms, which may provide further support to the idea that the subcortical gray matter lesions may represent a process specific to LOD. Therefore, the individuals in the present study with Alzheimer's disease and LOD may have a “double dose” of neuropathology—one that is primarily cortical (Alzheimer's disease) and the other subcortical (LOD).

### 4.3. Limitations

#### 4.3.1. Technological

 This study has several limitations. First is the use of CT instead of MRI. CT has better specificity than MRI for white matter changes of clinical significance—95.5% versus 68.2%, respectively, for frontal white matter changes, and 95.5% versus 63.3% for whole brain white matter changes [43]. This was supported by the high interrater reliability for bilateral WMH ratings; whereas, the basal ganglia-thalamic hypodensity ratings were relatively less reliable. However, CT is known for being less sensitive in detecting subtle microvascular ischemic changes, especially in individuals with Alzheimer's disease, the diagnosis assigned to the majority of this study's participants. In one study comparing MRI to CT neuroimaging of individuals with Alzheimer's disease, white matter changes in the frontal lobe were evident in seven out of the 22 participants using MRI, but only one out of the same 22 participants using CT. Additionally, sensitivity to mild frontal lobe WMH was only 31.0% for CT imaging versus 75.9% for MRI [43]. Thus, it is possible that the white matter changes found by other studies are too subtle to be detected by CT. Nonetheless, white matter changes in the present study were found to increase with age, as would be expected, and to be more common in vascular dementia than in other dementias.

#### 4.3.2. Cross-Sectional Study Design

A major limitation inherent in the study design is that CT data were available for only one point in time and on average five years after the age of dementia onset. Due to our lack of premorbid CT data, we can make no causal statements regarding depression and the presence of white or gray matter hypodensities, nor can we make any statements about change over time relative to progression of dementia. At the same time, somewhat surprisingly, there was not a significant relationship between duration of dementia and amount of white matter hypodensities in either the frontal lobe or subcortical white matter. For striatal hypodensities, there was no relationship when the entire sample was examined as a whole. Taken together with the greater amount of striatal hypodensities in the LOD/Alzheimer's disease group compared to nondepressed Alzheimer's disease group, this association, although cross-sectional, lends further support to the idea that there may be two separate neurodegenerative processes occurring among the individuals with comorbid Alzheimer's disease and LOD.

#### 4.3.3. Depression Assessment

 Another limitation was that the methods for ascertaining a history of depression were more sensitive to detecting recent episodes. Indeed, in our sample only 8.2% had a depressive episode prior to age 60, which is well below the estimated lifetime prevalence rate of 19.5% for major depression in Swedish twins [44]. Thus individuals in the late-onset group may have been misclassified due to unreported early episodes of depression, although they would have been correctly classified in analyses of late-life depression. In addition, findings from a recent study of the population from which the present study sample was drawn indicated that depression closer in time to dementia onset, and not earlier life depression, was associated with an increased risk of dementia [3]. Therefore, it is also possible that there was differential survival in individuals with early-onset depression, such that they were not as likely as individuals with LOD to develop dementia, and thus were underrepresented as a whole in this sample.

Another limitation stems from the fact that there is no consensus as to what age constitutes early-onset versus late-onset and late-life depression. A recent meta-analysis [45] of WMH in late-life depression noted that there was substantial variability across studies in the age cutoffs used to define late-onset depression, with ages ranging from 45 to 65 years of age. Whereas Alexopoulos et al. [29] proposed that vascular depression be defined by an age of onset after 65 years, Krishnan et al. [46] suggested a definition using an age cutoff of 50 years. Devanand et al. [47] recommended an age cutoff of 60 years because differences in cardiovascular risk factors between an earlier versus a later age of depression onset are greatest at this point.

Another reason for the differences in findings between our study and the findings of some previous studies of depression in those without dementia could be the nature of our depression measures. Our depression measures were dichotomous, and there was no way to distinguish between levels of depression severity. As suggested by previous studies of late-life depression (e.g., [21, 23]) white matter changes are related to the severity of late-onset depression, with differences between non-depressed and late-onset depression groups being evident only when the level of depression is relatively severe. Therefore, as the late-onset depression group in our study likely represented a wide range of depression severity (and because there were not enough individuals in our sample for a separate analysis with depression severe enough to require hospitalization), differences between the non-depressed and LOD groups could have been obscured due to the number of individuals with milder levels of depression.

## 5. Conclusions

In support of the fronto-striatal hypothesis of depression, the results of the present study indicated that among individuals with Alzheimer's disease, there was a significant relationship between late-onset depression and a greater amount of striatal hypodensities (gray matter hypodensities in the caudate nucleus and lentiform nucleus) compared to people with no depression. As subcortical changes are not typically associated with Alzheimer's disease, the greater amount of striatal hypodensities evident in the individuals with Alzheimer's disease and LOD may represent a neuropathological process that is distinct from the neurodegenerative changes caused by Alzheimer's disease. We did not find an association between LOD/LLD and white matter hypodensities in the frontal deep white matter or subcortical white matter. Other studies in individuals without dementia finding such an association have typically used MRI. Thus, associated white matter changes may have been too subtle to be detected by CT imaging. However, as prior studies were among individuals without dementia, it is equally likely that the neurological damage in the white matter caused by dementia subsumes the neuropathology of late-onset depression, such that the two neuropathologies become indistinct with respect to white matter. Thus, for older adults with dementia and late-life depression, there are important public health implications for identifying the pathophysiologic mechanisms linking these two conditions. Given that many treatments for depression are mediated by neurobiological factors, the efficacy of these treatments may be compromised in individuals with neuroanatomical abnormalities. An understanding of the unique brain changes associated with dementia versus those associated with LOD may elucidate avenues for treatments with better efficacy in older adults.

## Figures and Tables

**Table 1:** Sample demographics and covariates for all dementia and alzheimer's disease only.

*N*	All dementia	Alzheimer's disease
	182	127
Age at CT scan (SD)	80.6 (6.7)	80.8 (6.9)
Duration of dementia (SD)	5.2 (4.2)	5.1 (4.2)
Female, *n* (%)	116 (63.7)	86 (67.7)
Education, years (SD)	7.4 (2.4)	7.5 (2.6)
Monozygotic, *n* (%)	51 (28.0)	40 (31.5)
Hypertension, *n* (%)	153 (84.1)	104 (81.9)
Diabetes, *n* (%)	43 (23.6)	24 (18.9)
Arrhythmia, *n* (%)	36 (19.8)	25 (19.7)
PAD, *n* (%)	4 (2.2)	2 (1.6)
CAD, *n* (%)	59 (32.4)	38 (30.0)
TIA, *n* (%)	14 (7.7)	6 (4.7)
Any CVD, *n* (%)	166 (91.2)	113 (89.0)

PAD: peripheral artery disease; CAD: coronary artery disease; TIA: transient ischemic attack; CVD: cerebrovascular disease.

**Table 2:** Sample demographics by depression group.

*N*	No Depression	LOD	*P* value*
	132	36	

Age at CT scan (SD)	81.1 (6.1)	81.0 (7.0)	n.s.
Age at dementia onset (SD)	75.9 (6.9)	76.9 (6.7)	n.s.
Dementia duration, years (SD)	5.2 (4.2)	4.2 (3.5)	n.s.
% Female	63.6	63.9	n.s.
Education, years (SD)	7.5 (2.4)	7.4 (2.8)	n.s.
Monozygotic, *n* (%)	33 (25.0)	15 (41.7)	**0.05**
Hypertension, *n* (%)	113 (85.6)	30 (83.3)	n.s.
Diabetes, *n* (%)	32 (24.2)	6 (16.7)	n.s.
Arrhythmia, *n* (%)	25 (18.9)	8 (22.2)	n.s.
PAD, *n* (%)	3 (2.3)	1 (2.8)	n.s.
CAD, *n* (%)	41 (31.1)	16 (44.4)	**0.13**
TIA, *n* (%)	14 (10.7)	0 (0.0)	**0.04**

Associations significant at *P* < 0.15 are bolded.

LOD: late-onset depression; PAD: peripheral artery disease; CAD: coronary artery disease; TIA: transient ischemic attack.

*Group comparisons by ANOVA for continuous variables and by chi-square for categorical variables.

**Table 3:** Risk of confluent frontal lobe deep white matter hypodensities in late-onset depression compared with No depression for all dementia and alzheimer's disease.

	No depression		Late-onset depression		
	−Con WMH	+Con WMH	−Con WMH	+Con WMH	OR*
	*N* (%)	*N* (%)	*N* (%)	*N* (%)	(95% CI)

All dementia	97 (73.5)	35 (26.5)	23 (63.9)	13 (36.1)	1.58 (0.72, 3.46)
AD only	73 (76.8)	22 (23.2)	16 (64.0)	9 (36.0)	1.83 (0.70, 4.79)

−ConWMH: Confluent frontal deep white matter hypodensities absent; +ConWMH: Confluent frontal deep white matter hypodensities present; OR: Binary logit odds ratio estimate, controlling for gender; CI: confidence interval; AD: Alzheimer's disease.

*Late-onset depression group compared to no depression group.

**Table 4:** risk of frontal lobe deep white matter hypodensities in late-onset depression compared with no depression for all dementia and Alzheimer's disease.

	No depression		Late-onset depression		
	−WMH	+WMH	−WMH	+WMH	OR
	*N* (%)	*N* (%)	*N* (%)	*N* (%)	(95% CI)*

All dementia	75 (56.8)	57 (43.2)	22 (61.1)	14 (38.9)	0.96 (0.44, 2.08)
AD only	59 (62.1)	36 (37.9)	16 (64.0)	9 (36.0)	0.88 (0.35, 2.25)

−WMH: frontal deep white matter hypodensities absent; +WMH: frontal deep white matter hypodensities present; OR: binary logit odds ratio estimate, controlling for gender; CI: confidence interval; AD: Alzheimer's disease.

*Late-onset depression group compared to no depression group.

**Table 5:** risk of subcortical white matter hypodensities in late-onset depression compared with no depression for all dementia and Alzheimer's disease only.

	No depression				Late-onset depression				
Level of WMH*	0	1	2	3	0	1	2	3	OR (95% CI)**

All dementia	62 (47.0)	16 (12.1)	25 (18.9)	29 (22.0)	17 (47.2)	3 (8.33)	9 (25.0)	7 (19.4)	0.97 (0.48, 1.92)
AD only	48 (50.5)	11 (11.6)	18 (18.9)	18 (18.9)	12 (48.0)	2 (8.0)	5 (20.0)	6 (24.0)	1.16 (0.48, 2.58)

OR: ordinal cumulative logit odds ratio estimate, controlling for age at time of CT scan; CI: confidence interval; AD: Alzheimer's disease.

Level of WMH: 0 = none, 1 = one discrete hypodensity, 2 = more than one discrete hypodensity, 3 = confluent hypodensities.

*All numbers for Level of WMH given in terms of *N* (%). **Late-onset depression group compared to no depression group.

**Table 6:** number of striatal hypodensities in late-onset depression compared with no depression for all dementia and Alzheimer's disease only.

	No depression	LOD	No depression versus LOD*
*All dementia*			
*N*	132	36	
Striatum, mean (SD)	0.48 (0.96)	0.86 (1.53)	0.07
*Alzheimer's disease only*			
*N*	95	25	
Striatum, mean (SD)	0.42 (0.92)	0.96 (1.72)	**0.03**

LOD: late-onset depression.

*P value.
